# Hypopituitarism with secondary adrenocortical insufficiency and arginine vasopressin deficiency due to hypophysitis after COVID-19 vaccination: a case report

**DOI:** 10.1186/s12902-024-01582-9

**Published:** 2024-05-20

**Authors:** So Watanabe, Yoshiaki Tamura, Kazuhito Oba, Saori Kitayama, Motoya Sato, Remi Kodera, Kenji Toyoshima, Yuko Chiba, Atsushi Araki

**Affiliations:** Department of Diabetes, Metabolism, and Endocrinology, Tokyo Metropolitan Institute for Geriatrics and Gerontology, 35-2 Sakaecho, Itabashi-Ku, Tokyo, 173-0015 Japan

**Keywords:** COVID-19 vaccination, Hypopituitarism, ASIA, Secondary adrenocortical insufficiency, Arginine vasopressin deficiency

## Abstract

**Background:**

Although vaccination against coronavirus disease (COVID-19) has several side effects, hypopituitarism due to hypophysitis has rarely been reported.

**Case presentation:**

An 83-year-old healthy woman, who had received her fourth COVID-19 vaccine dose 2 days before admission, presented to the emergency department with difficulty moving. On examination, impaired consciousness (Glasgow Coma Scale: 14) and fever were observed. Computed tomography and magnetic resonance imaging of the head revealed swelling from the sella turcica to the suprasellar region. Her morning serum cortisol level was low (4.4 μg/dL) and adrenocorticotropic hormone level was normal (21.6 pg/mL). Central hypothyroidism was also suspected (thyroid stimulating hormone, 0.46 μIU/mL; free triiodothyronine, 1.86 pg/mL; free thyroxine, 0.48 ng/dL). Secondary adrenocortical insufficiency, growth hormone deficiency, delayed gonadotropin response, and elevated prolactin levels were also observed. After administration of prednisolone and levothyroxine, her consciousness recovered. On the 7th day of admission, the patient developed polyuria, and arginine vasopressin deficiency was diagnosed using a hypertonic saline test. On the 15th day, the posterior pituitary gland showed a loss of high signal intensity and the polyuria resolved spontaneously. On the 134th day, the corticotropin-releasing hormone loading test showed a normal response; however, the thyrotropin-releasing hormone stimulation test showed a low response. The patient’s disease course was stable with continued thyroid and adrenal corticosteroid supplementation.

**Conclusions:**

Herein, we report a rare case of anterior hypopituitarism and arginine vasopressin deficiency secondary to hypophysitis following COVID-19 vaccination.

## Background

Coronavirus disease (COVID-19) vaccines are produced by targeting the S protein of the virus. They have been reported to be effective in preventing COVID-19 and severe COVID-19, as well as reducing the incidence of sequelae [1]. BNT162b2 (Pfizer/BioNTech) and mRNA-173 (Moderna) as mRNA vaccines, ChAdOx1 nCOV-19 (AstraZeneca) as an adenovirus vector, and NVX-CoV 2373 (Novavax) as a recombinant S protein, have been used in Japan. These vaccines can cause various side effects such as pain, swelling, fever, headache, and diarrhea [2]. Regarding the pituitary gland, pituitary apoplexy [3–5] and arginine vasopressin (AVP) deficiency [6–11] have rarely been reported after COVID-19 vaccination. Only three cases of hypophysitis and anterior pituitary hormone deficiency have been reported: low insulin-like growth factor [11], isolated adrenocorticotropic hormone (ACTH) deficiency [12], and anterior hypopituitarism [11, 13].

Herein, we present a rare case of panhypopituitarism due to hypophysitis after COVID-19 vaccination, in which symptoms improved with hormone replacement therapy and hormonal secretion improved partially during the clinical course.

## Case presentation

An 83-year-old previously healthy woman, with no growth or developmental abnormalities, presented to the emergency room with fever and immobility 2 days after her fourth dose of the COVID-19 vaccine (BNT16B2b2, an mRNA vaccine containing spike-protein mRNA), and was admitted to our hospital. She had previously received three doses of the same COVID-19 vaccine; however, after each session, she presented with only slight side effects such as pain, redness, and fever at the injection site, which disappeared within a few days. She also had hyperlipidemia, hyperuricemia, and an overactive bladder, for which she received rosuvastatin calcium, febuxostat, vibegron, and vonoprazan fumarate for an extended period. She was a social drinker and did not smoke.

**Fig. 1:**
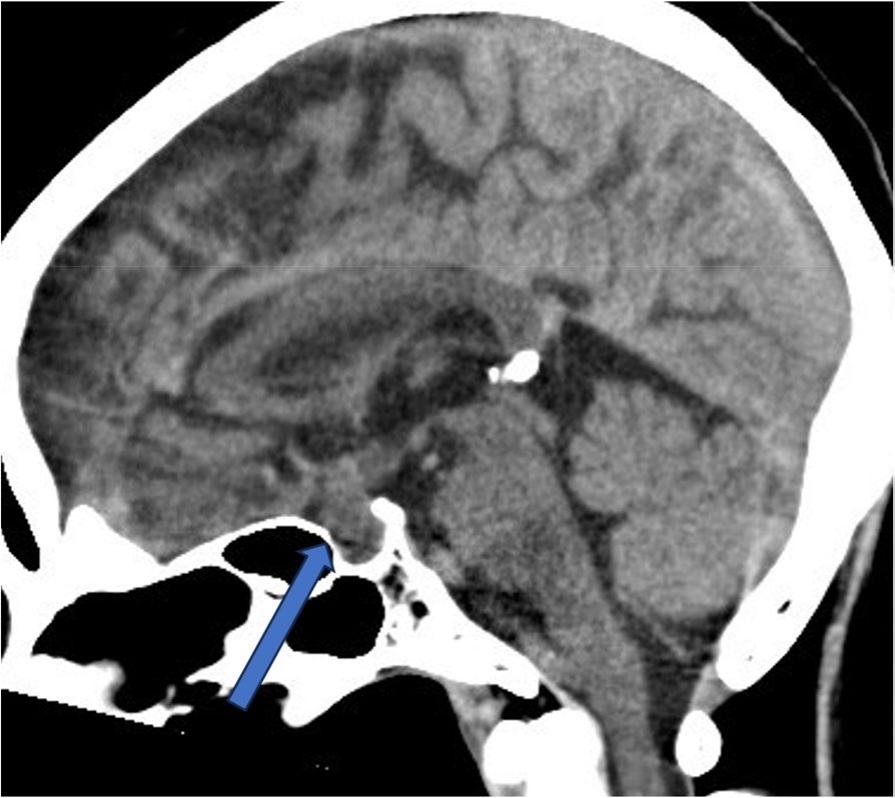
Computed tomography (CT) sagittal image of the brain (2nd day of admission). The arrow shows the enlargement of the sella turcica to the suprasellar region

**Fig. 2:**
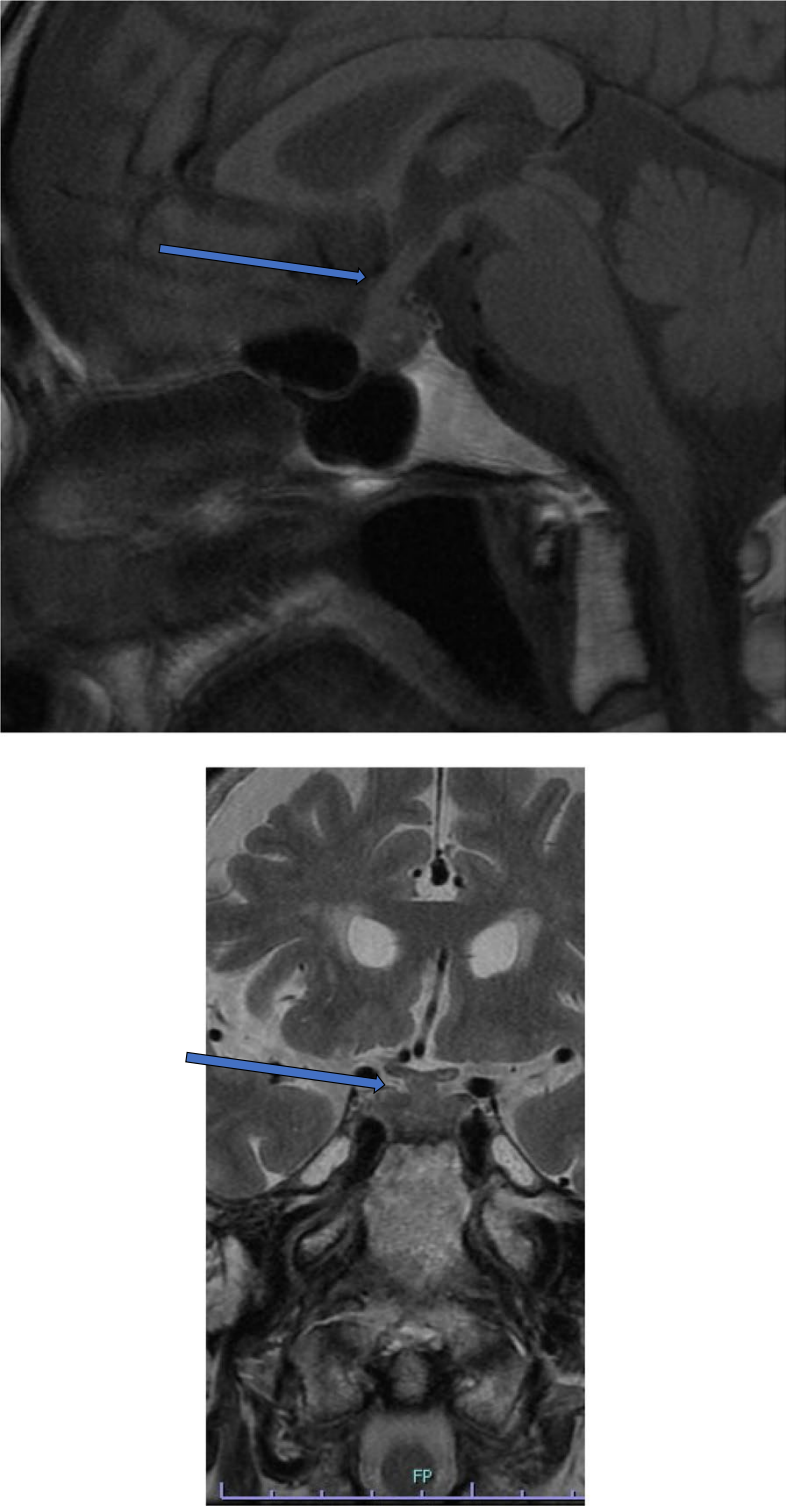
T1-weighted sagittal and T2- weighted coronal MRI of the brain (3rd day of admission). The arrow shows a mass-like lesion from the sella turcica to the suprasellar region and a thickened pituitary stalk. MRI, Magnetic resonance imaging

On examination, the patient had impaired consciousness (Glasgow Coma Scale: 14 [E4V4M6]) and fever (37.9 ℃). Her pulse rate was 92 beats/min, blood pressure was 110/70 mmHg, height was 149.1 cm, and body weight was 53.2 kg. We observed discharge and skin redness around both eyelids. No abnormalities were observed in the thyroid gland, chest, or abdomen. Laboratory tests revealed an elevated inflammatory response (C-reactive protein, 2.89 mg/dL; Table 1). Computed tomography (CT) of the head revealed enlargement of the sella turcica with extension into the suprasellar region (Fig. 1). Magnetic resonance imaging (MRI) of the head also revealed a large-for-age pituitary gland and a thickened pituitary stalk (Fig. 2).

**Table 1:** Patient’s laboratory test results on admission

Test	Result		Reference range
Arterial blood gas (room air)			
pH	7.45		7.35 − 7.45
pO_2_	82.8	mmHg	76 − 94
pCO_2_	34.9	mmHg	35 − 46
HCO_3_-	23.7	mmol/L	21 − 26
Base excess	0.3	mmol/L	-2 − + 2
Blood count			
WBC	8310	/μL	3300 − 8600
Hb	14.9	g/dL	11.6 − 14.8
Plt	22.8	10^4^/μL	15.8 − 34.8
Biochemistry			
TP	6.6	g/dL	6.6 − 8.1
Alb	3.8	g/dL	4.1 − 5.1
CRP	2.83	mg/dL	≤ 0.14
T-bil	1.1	mg/dL	0.4 − 1.5
AST	23	U/L	13 − 30
ALT	13	U/L	7 − 23
LDH	177	U/L	124 − 222
ALP	49	U/L	38 − 113
γGTP	17	U/L	9 − 32
CK	125	U/L	41 − 153
BUN	10	mg/dL	8 − 20
Cre	1.35	mg/dL	0.46 − 0.79
Na	138	mEq/L	138 − 145
K	3.7	mEq/L	3.6 − 4.8
Cl	102	mEq/L	101 − 108
Ca	9.7	mg/dL	8.8 − 10.1
Plasma glucose	112	mg/dL	73 − 109
Morning Cortisol	4.4	μg/dL	6.4 − 21.0
ACTH	21.6	pg/mL	7.2 − 63.3
TSH	0.46	μIU/mL	0.38 − 4.31
fT3	1.86	pg/mL	2.10 − 3.80
fT4	0.48	ng/dL	0.82 − 1.63
Prolactin	55.45	ng/mL	6.12 − 30.54
IgG	1,113	mg/dL	861 − 1,747
IgG4	41.9	mg/dL	11 − 121
ACE	9.4	IU/L	7.7 − 29.4

*WBC* White blood cell, *Plt* Platelet, *TP* Total protein, *Alb* Albumin, *CRP* C-reactive protein, *IgG* Immunoglobulin G, *ACE* Angiotensin converting enzyme

The next morning, her serum cortisol was low (4.4 μg/dL) and adrenocorticotrophic hormone (ACTH) level was normal (21.6 pg/mL). All thyroid hormones including thyroid-stimulating hormone (TSH, 0.46 μIU/mL); free triiodothyronine (1.86 pg/mL); and free thyroxine (0.48 pg/mL) were low; hence, central hypothyroidism was suspected. Her prolactin levels were elevated (55.45 ng/mL). Immunoglobulin G (IgG), IgG4, and angiotensin-converting enzyme levels were within the normal ranges (Table 1).

**Fig. 3:**
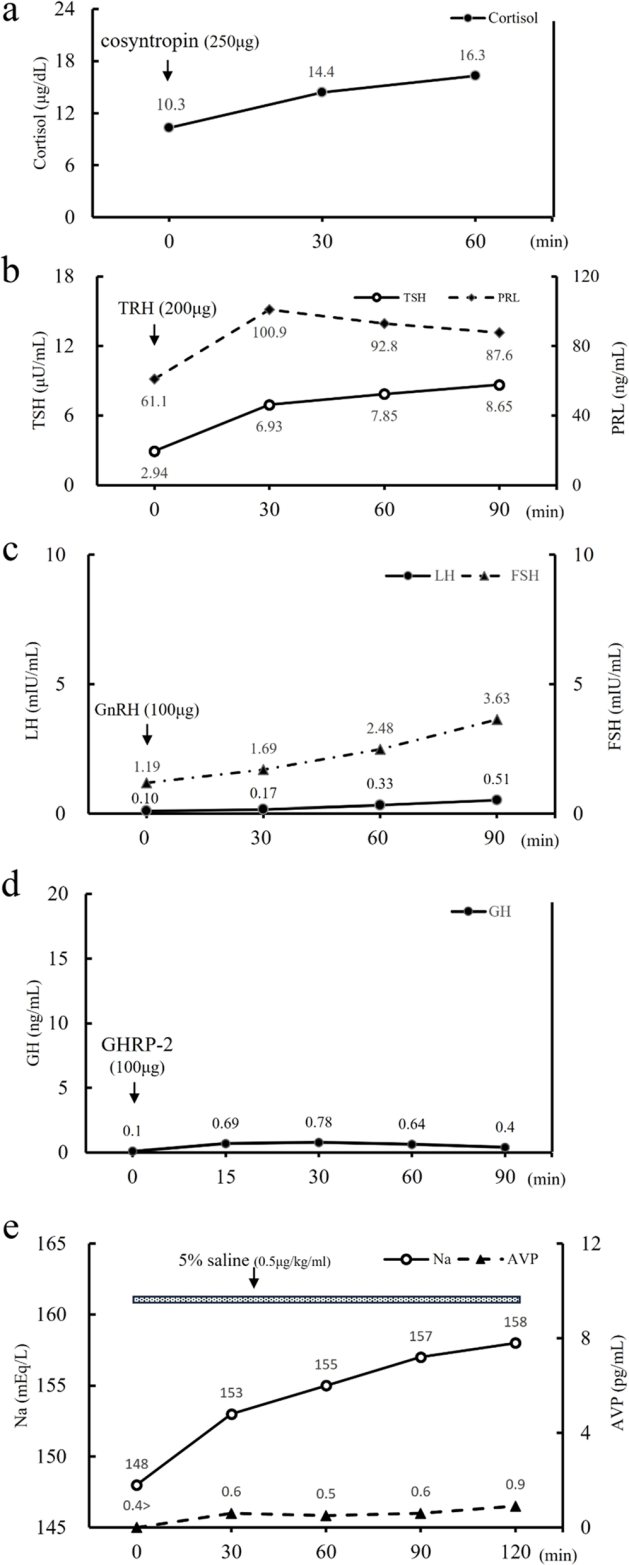
Pituitary provocation tests on admission. **a**, **b**, **c**, **d** represent the results after **a**: CRH (250 μg, intravenous [i.v.]), **b**: TRH (200 μg, i.v.), **c**: GnRH (100 μg, i.v.), and **d**: GHRP-2 (100 μg, i.v.) load. **e**: Result of hypertonic saline test. CRH, corticotrophin-releasing hormone; TRH, thyrotropin-releasing hormone; TSH, thyroid-stimulating hormone; PRL, prolactin; GnRH, gonadotropin-releasing hormone; LH, luteinizing hormone; FSH, follicle-stimulating hormone; GHRP-2, growth hormone-releasing peptide-2; GH, growth hormone; AVP, arginine vasopressin

Hormone replacement therapy comprising prednisolone (30 mg/day) and levothyroxine (12.5 μg/day) was administered, after which her consciousness improved. On the 4th day, the cosyntropin stimulation test was performed and secondary adrenal insufficiency was suspected as the peak value was < 18 µg/dL (Fig. 3a). Thyrotropin-Releasing hormone (TRH) and gonadotropin-releasing hormone loading tests on the 8th day also revealed growth hormone deficiency and delayed responses to TSH, luteinizing hormone, and follicle-stimulating hormone (Fig. 3b–d). Growth Hormone (GH)-releasing peptide-2 testing performed on the same day revealed a peak value below 9 µg/L, hence the diagnosis of adult GH deficiency [14]. These anterior pituitary hypofunctions suggested panhypopituitarism.

**Fig. 4:**
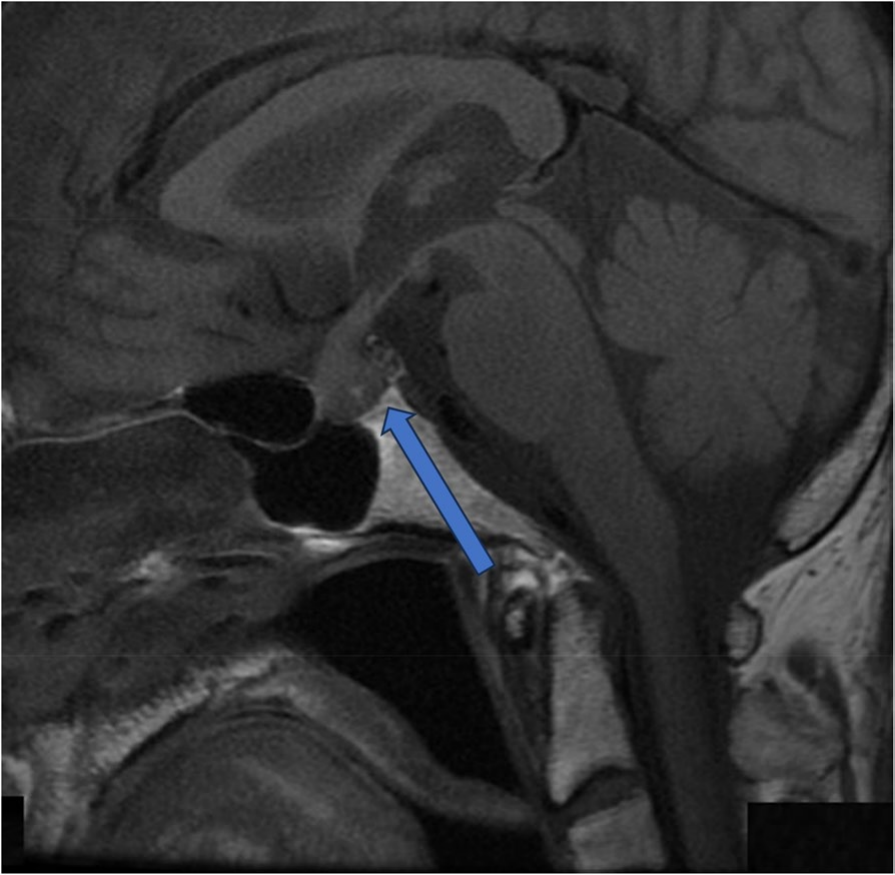
T1-weighted sagittal magnetic resonance imaging (MRI) of the pituitary (10th day of admission). The arrow shows the disappearance of the bright spots in the posterior pituitary gland

On the 7th day of hospitalization, the patient developed polyuria (4150 mL/day). AVP deficiency was diagnosed using a hypertonic saline test (Fig. 3e). Her MRI showed that the high signal intensity (bright spots) in the posterior pituitary lobes had disappeared (Fig. 4). Polyuria resolved spontaneously on the 15th day. Replacement therapy comprising thyroid and adrenal corticosteroid hormones was continued. Growth hormone replacement was not initiated due to the absence of symptoms. The patient was discharged on the 19th day.

**Fig. 5:**
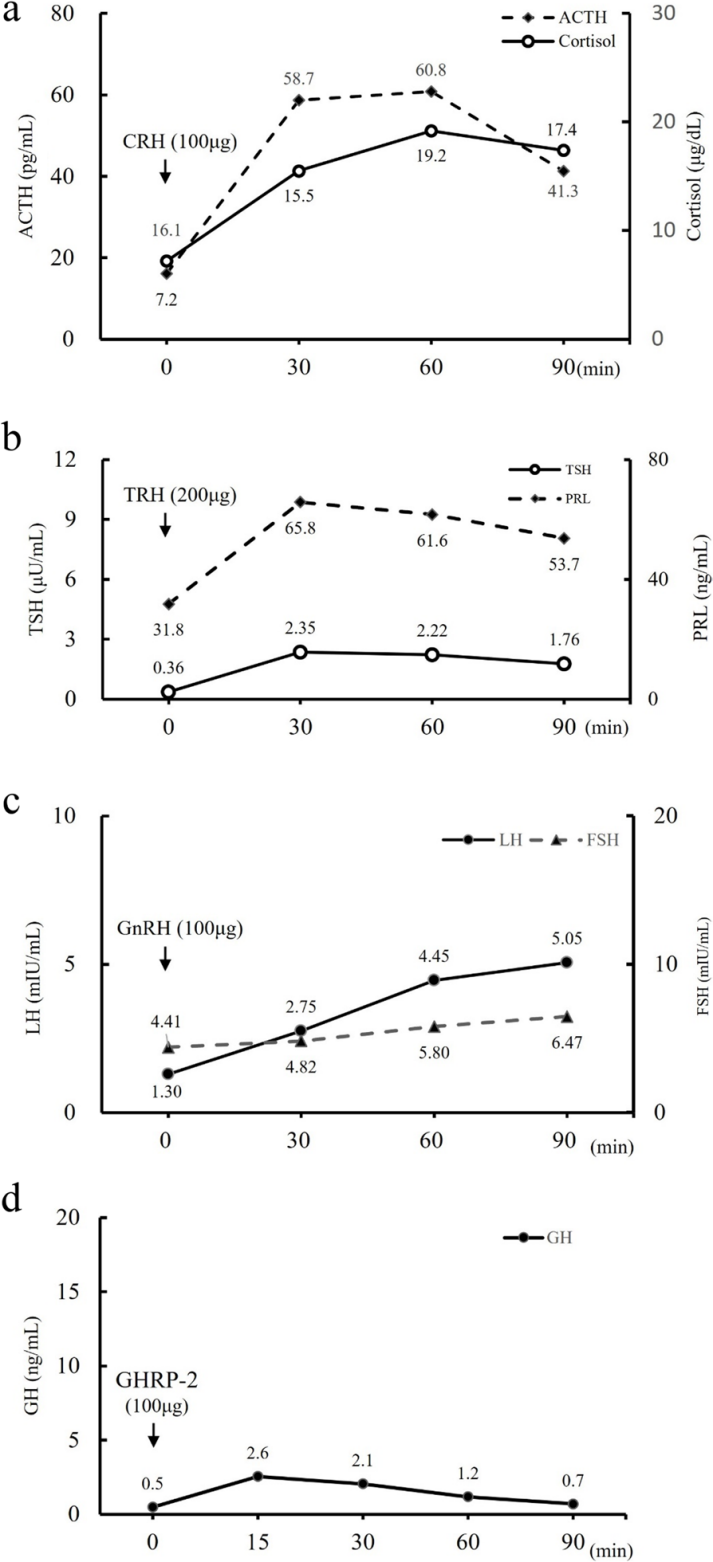
Pituitary provocation tests after 3 months. **a**, **b**, **c**, **d** represent the results after **a**: CRH (250 μg, i.v.), **b**: TRH (200 μg, i.v.), **c**: GnRH(100 μg, i.v.), and **d**: GHRP-2 (100 μg, i.v.) load. CRH, corticotropin-releasing hormone; ACTH, adrenocorticotropic hormone; TRH, thyrotropin-releasing hormone; TSH, thyroid-stimulating hormone; PRL, prolactin; GnRH, gonadotropin-releasing hormone; LH, luteinizing hormone; FSH, follicle-stimulating hormone; GHRP-2, growth hormone-releasing peptide-2; GH, growth hormone

**Fig. 6:**
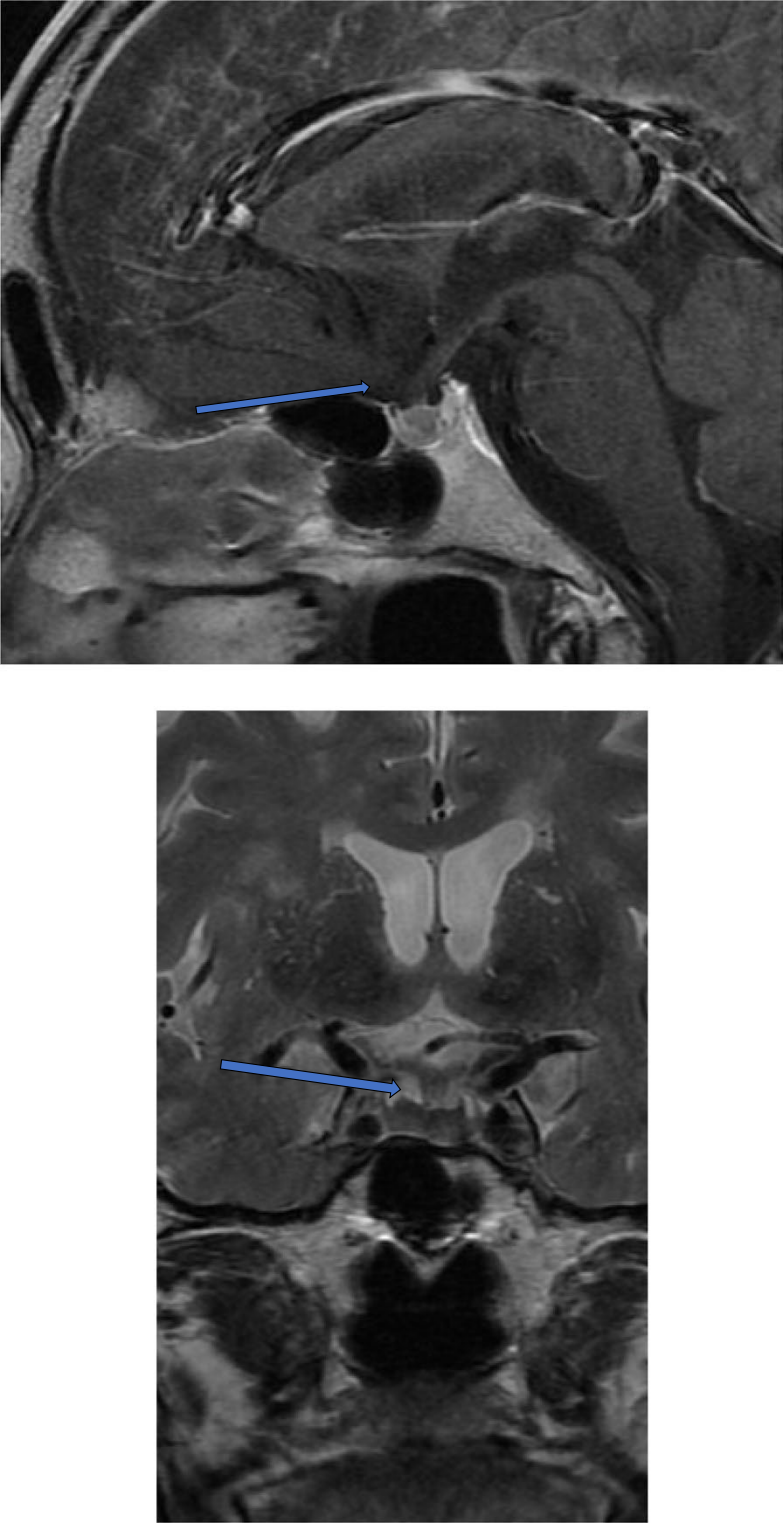
Contrast-enhanced T1-weighted sagittal and T2- weighted coronal MRI of the brain (137th day after admission). The arrow shows improvement of edematous changes in the pituitary stalk. MRI, magnetic resonance imaging

On the 134th day, the patient was readmitted for evaluation of endocrine function. The cosyntropin stimulation test showed a normal response; however, the TRH stimulation test had a low response. Luteinizing hormone-releasing hormone and GH-releasing peptide-2 load tests also showed low responses (Fig. 5). Contrast-Enhanced MRI of the pituitary gland showed further improvement in the edematous changes in the pituitary stalk (Fig. 6). As hormonal secretion partially improved, supplementation with thyroid and adrenal corticosteroid hormones was continued. The patient’s condition stabilized after hormone supplementation.

## Discussion

This was a rare case of hypopituitarism possibly due to hypophysitis 2 days after COVID-19 vaccination. It is well known that COVID-19 vaccination can induce endocrine dysfunctions such as subacute thyroiditis, Graves' disease [15], adrenal crisis [16, 17], and type 1 diabetes mellitus [18]. Pituitary disorders after vaccination, including pituitary apoplexy [3–5], AVP deficiency [6–11], isolated ACTH deficiency [12], and anterior hypopituitarism [11, 13], have rarely been reported.

Hypophysitis is defined as a primary or secondary inflammation of the pituitary gland. Although a rare cause, accounting for 1.6% of all cases of hypopituitarism, the incidence of hypopituitarism is on the rise with the increased use of checkpoint inhibitors in cancer treatment [19]. Patients with hypopituitarism typically present with headaches and deficiencies in anterior and/or posterior pituitary hormones. In contrast to the two previous cases of hypophysitis with secondary adrenal insufficiency (Table 2), this case was characterized by the onset of difficulty in moving and impaired consciousness rather than gastrointestinal symptoms. Various types of hypophysitis are associated with autoimmune diseases, IgG4-related diseases, sarcoidosis, and immune checkpoint inhibitors [20]. The differential diagnoses of hypophysitis are pituitary tumors, including metastases; pituitary apoplexy; and lymphoproliferative disease.

**Table 2:** Review of previous case reports of hypopituitarism associated with COVID-19 vaccination

Authors	Age, sex	Diagnosis	Vaccine	Symptoms	Onset after vaccination	Thyrotropin Axis	Gonadal Axis	Corticotropin Axis	Lactotropin Axis	AVP-D*	Images	Treatment	Clinical course	Replacement therapy
***Cases of arginine vasopressin deficiency***														
Ishay A, et al	59 y, F	AVP-D	BNT162b2	Polyuria, thirst, fatigue, weight loss	8 weeks	N	N	N	N	Y	Thickened pituitary stalk, disappearance of bright spots in posterior lobe	Desmopressin	Symptoms improved	Continued (18 months)
Bouça B, et al	37 y, M	AVP-D	BNT162b2	Polyuria, thirst	7 days	N	N	N	N	Y	Disappearance of bright spots in posterior lobe	Desmopressin	Symptoms improved	Continued (2 months)
Partenope C, et al	16 y, M	AVP-D	BNT162b2	Polyuria, thirst, fatigue	a few days	N	N	N	N	Y	Thickened pituitary stalk, disappearance of bright spots in posterior lobe	Desmopressin	Symptoms improved	Continued (3 months)
Matsuo T, et al	74y, F	AVP-D, multiple sclerosis	BNT162b2	Polyuria, thirst, fatigue, gait disturbance, intentional tremor	1 month	N	N	N	N	Y	Thickened pituitary stalk, disappearance of bright spots in posterior lobe	Desmopressin	Symptoms improved	N/M
Ach T, et al	54 y, F	AVP-D	ChAdOx1	Polyuria, thirst weight loss	3 days	N	N	N	N	Y	Thickened pituitary stalk	Desmopressin	N/M	N/M
Ankireddypalli AR, et al	48 y, F	HP	BNT162b2	Polyuria, thirst, headache, fatigue, polyarthralgia	2 days	N	Hypo-gonadism	N	N	Y	Thickened pituitary stalk, partially empty sella	Desmopressin	Symptoms partially improved	Continued
***Cases of anterior pituitary hormone deficiency***														
Ankireddypalli AR, et al	48 y, F	HP	BNT162b2	Amenorrhea	2 days	N	Hypo-gonadism	N	N	Y	Thickened pituitary stalk, partially empty sella	N	Menstrual recovery	N
Murvelashvili N, Tessnow A	51 y, M	HP	mRNA-173	Nausea, vomiting, abdominal pain, decreased libido, erectile dysfunction	3 days	Hypo-thyroidism	Hypo-gonadism	Adrenocortical insufficiency	N	N	Enlargement of pituitary gland	Glucocorticoid Levothyroxine	Testosterone normalized Pituitary gland enlargement diminished with empty sella	Continued (1 month)
Morita S, et al	31 y, M	IAD	BNT162b2	Fever, headache, malaise, nausea, diarrhea	1 day	N	N	Adrenocortical insufficiency	Hyper prolactinemia	N	Atrophy of the anterior pituitary lobe	Glucocorticoid	Symptoms improved	Continued (3 months)
Current case	83 y, F	HP	BNT162b2	Fever, unable to move, impaired consciousness	2 days	Hypo-thyroidism	Hypo-gonadism	Adrenocortical insufficiency	Hyper prolactinemia	Y	enlargement of pituitary gland Thickened pituitary stalk, disappearance of bright spots in posterior lobe	Glucocorticoid Levothyroxine	Symptoms, Corticotropin axis and AVP-D* improved Pituitary stalk edema improved	Continued (140 days)

*F* Female, *M* Male, *AVP-D* arginine vasopressin deficiency, *HP* hypophysitis, *IAD* isolated ACTH deficiency, *N/M* not mentioned, *ACTH* adrenocorticotrophic hormone

MRI findings are characterized by moderate pituitary gland enlargement, homogeneous contrast enhancement, a thickened pituitary stalk, the absence of posterior pituitary bright spots, and an empty sella turcica after the inflammatory process [21]. In the present case, the presence of an enlarged pituitary gland with an edematous and thickened pituitary stalk supported the diagnosis of hypophysitis. The spontaneous improvement in pituitary imaging and hormone levels over time suggests transient immune abnormalities and inflammation after vaccination. Blood tests and whole-body CT showed no evidence of sarcoidosis, IgG4-related diseases, or other diseases that could cause pituitary inflammation. There was also no history of use of immune checkpoint inhibitors or other medications. In addition, pituitary inflammation following vaccination has been previously reported to develop within 5 days of vaccination [11]. In our patient, it developed two days after vaccination, suggesting an association with vaccination.

To the best of our knowledge, only eight cases of hypopituitarism associated with COVID-19 vaccination have been reported (six cases of AVP deficiency and three cases of anterior pituitary hormone deficiency; Table 2). In contrast to previous studies, our case was an older patient with at least three pituitary hormone deficiencies, later onset of transient AVP deficiency symptoms that did not require treatment, and partial hormonal recovery and improvement of imaging findings during the clinical course.

Although the mechanism by which hypophysitis occurs after vaccination is unknown, several hypotheses have recently been proposed, including the possibility of molecular mimicry, vaccinia adjuvants, and innate immune activation [22]. In individuals with certain genetic backgrounds, cross-reactivity may occur due to similarities between vaccine components and their own proteins, called molecular mimicry. Reactions to self-antigens can cause autoimmune disease. In addition, adjuvants in vaccines may cause autoimmune/inflammatory syndrome (ASIA) [23], which is defined as exposure to an external stimulus, development of symptoms suggestive of an autoimmune syndrome, and improvement after removal of the triggering substance. The mRNA vaccine, BNT162b2, does not contain insoluble aluminum but lipid nanoparticles for structural stabilization [24], which may be responsible for the adjuvant activity that causes ASIA [25]. The mRNA and DNA vaccines themselves can stimulate Toll-like receptors 7 and 9, which lead to autoimmune diseases as a result of elevated type 1 interferon and increased immunoglobulin via age-related B-cell proliferation [26]. AVP deficiency and hyperprolactinemia may be partially due to thickening of the pituitary stalk, which may have resulted in impairment of AVP and dopamine, a prolactin-suppressing hormone [27]. Therefore, the AVP deficiency improved with amelioration of the pituitary pattern thickening.

The prognosis of hypopituitarism after COVID-19 vaccination remains unclear. Drug-induced hypopituitarism, caused by immune checkpoint inhibitors such as cytotoxic T-lymphocyte antigen-4, often requires long-term hormone replacement therapy with glucocorticoids [28]. Lymphocytic hypopituitarism requires at least one type of hormone replacement therapy in 73% of patients [29]. The course of ASIA is generally good, with a good response to steroids and other medications; however, the response to drug withdrawal is unknown [22]. There is no description of the recovery of hormone levels in the three previous cases of hypopituitarism induced by COVID-19 vaccines [11–13]. In some cases of AVP deficiency, long-term replacement of vasopressin is necessary [6–8, 11]. Nevertheless, it is also reported that in about half cases of thyroid disorder, at least partial recovery was observed within 5 months [11], indicating a relatively better prognosis in ASIA than other etiologies. In our case, hypopituitarism occurred within a short time after vaccination, the symptoms of AVP deficiency and pituitary MRI imaging spontaneously improved, and the ACTH-cortisol system normalized. All these facts support the fact that the endocrine abnormalities in these patients were due to hypophysitis related to COVID-19 vaccination, probably via an ASIA mechanism. However, because of a persistently low response to the TRH stimulation test, the patient continued to receive cortisol and thyroid hormone supplementation. Further hormonal evaluations during follow-up should be conducted to determine whether the recovery time may differ between endocrine organs and whether these hormone replacements should be continued.

## Conclusions

Herein, we presented a rare case of hypopituitarism due to hypophysitis after COVID-19 vaccination, which was followed by partial improvement of hypopituitarism. In cases of impaired consciousness after COVID-19 vaccination, it may be important to consider hypophysitis in the differential diagnosis. However, as COVID-19 vaccination has been shown to reduce the risk of disease severity, and cases of hypopituitarism as a side effect are rare, the benefits outweigh the risks [30]. Hence, the possibility of hypopituitarism as an adverse reaction to vaccination should not influence vaccination decisions. Even high-risk individuals should be appropriately vaccinated.

## Data Availability

The datasets used in this case report are available from the corresponding author upon request.

## Abbreviations

ACTH: Adrenocorticotropic hormone
ASIA: Adjuvant-associated autoimmune/inflammatory syndrome
AVP: Arginine vasopressin
COVID-19: Coronavirus disease
CT: Computed tomography
GH: Growth hormone
MRI: Magnetic resonance imaging
TRH: Thyrotropin-releasing hormone
TSH: Thyroid stimulating hormone
